# Editorial: Genome editing for climate change adaptation in agriculture: innovations, applications, and regulatory considerations

**DOI:** 10.3389/fgeed.2025.1711767

**Published:** 2025-10-20

**Authors:** Koppolu Raja Rajesh Kumar, Prashant Kumar Singh

**Affiliations:** ^1^ Department of Biotechnology, Indira Gandhi National Tribal University, Amarkantak, Madhya Pradesh, India; ^2^ Department of Biochemistry, University of Lucknow, Lucknow, Uttar Pradesh, India

**Keywords:** CRISPR/Cas9 genome editing, crop improvement, climate-resilient crops, gRNA design, *in planta* transformation, virus-induced genome editing, biosafety, transgene-free genome editing

Human-induced climate change has unequivocally altered the global climate system, with surface temperatures rising by approximately 1.1 °C above pre-industrial levels during 2011–2020, and warming occurring more rapidly over land than oceans. This rise is already manifesting in widespread changes to weather and climate extremes across all regions, including more frequent and intense heatwaves, droughts, heavy precipitation and flooding (IPCC, 2023). Agriculture, in particular, is highly vulnerable to these changes, as climate-driven increases in drought and flooding threaten crop productivity, food security, and rural livelihoods. Moreover, the increasing frequency and intensity of climate extremes are expected to heighten the probability of concurrent yield losses across major food-producing regions (Bezner Kerr et al., 2022). While adaptation strategies such as water management, agroforestry, and the development of improved cultivars are being implemented, the scale and urgency of the climate challenge necessitate innovative solutions that can deliver resilient and sustainable agricultural systems. The integration of CRISPR/Cas9 genome editing into crop improvement programs represents one of the most significant advances in agricultural biotechnology, offering unprecedented precision to accelerate the breeding of climate-resilient varieties. Recent advances in genome editing technologies, together with the integration of genomics, phenomics, and artificial intelligence (AI)/machine learning (ML), offer significant opportunities for agricultural improvement. The four research articles published under the Research Topic highlight essential aspects of this transformative technology, from technical optimization to regulatory frameworks.

An essential requirement for successful genome editing is the design of highly specific guide RNAs (gRNAs), which direct the Cas9 nuclease to the intended genomic target. gRNA design is particularly challenging in wheat due to its complex allohexaploid genome, large genome size (∼17 Gb), and a high proportion of repetitive sequences. These factors, along with the prevalence of multi-gene families, complicate target recognition and increase the risk of off-target activity. Effective editing thus depends not only on selecting the correct gene target but also on optimizing gRNA design and addressing post-design considerations such as gRNA stability, binding efficiency, and functional activity. Addressing these challenges, the methods article by Singh et al. presents a consolidated and detailed workflow for efficient gene selection and gRNA design in wheat, using the *TaARE1* gene as an example. The authors systematically evaluate structural, physical, compositional, and free energy parameters through advanced bioinformatic tools to identify gRNAs with high on-target activity, and minimal off-target effects. Wheat, being a staple food crop for a significant portion of the global population, can benefit from this comprehensive methodology, which provides practical guidance for navigating the complexities of genome editing and developing improved varieties that are resilient to climate change.

Following the selection of the target gene and the design of recombinant vectors expressing gRNA and Cas9 or other RNA-guided endonucleases, a crucial subsequent step is the efficient delivery and transformation into plant cells using techniques such as *Agrobacterium tumefaciens*-mediated transformation or biolistic bombardment. However, this step represents a significant bottleneck in plant genome editing, as many crops require specialized transformation and regeneration protocols based on tissue culture. These processes are not only time-consuming and labor-intensive but also species-dependent. In contrast, *in planta* transformation methods have emerged as promising alternatives that bypass many of these challenges by targeting meristematic tissues, floral structures, or embryos directly, thereby reducing or eliminating the need for tissue culture steps. Additionally, success of novel approaches like *de novo* meristem induction, spray-on genome editing using carbon dot-recombinant plasmid complexes, and regenerative activity-dependent in-planta injection delivery (RAPID)—in some crop species further expand the possibilities for efficient gene editing in essential crops. In their review, Sebiani-Calvo et al. comprehensively discuss these *in planta* strategies, highlighting how such methods can overcome the limitations of tissue culture and broaden the applicability of genome editing for crop improvement. By eliminating the tissue-culture barrier, these methods can accelerate the creation of climate-resilient varieties.

While *in-planta* strategies have enabled genome editing without tissue culture, recent advances in viral vector–mediated approaches further expand the toolkit by eliminating the need for traditional transformation methods. For example, Qiao et al. (2025) demonstrated a virus-induced genome editing in tillers (ViGET) system that achieves high editing efficiency across diverse wheat genotypes. This was accomplished by engineering barley yellow striate mosaic virus (BYSMV) to simultaneously deliver the Cas9 nuclease and single guide RNA (sgRNA) directly into wheat plants without the need for tissue culture or stable transformation, enabling efficient, heritable, and transgene-free genome editing. This innovative approach opens new avenues for extending genome editing to other economically important crops that are difficult to genetically transform; however, its practical efficiency still requires systematic optimization and validation across diverse species to realize its full potential in crop improvement programs.

Many recent studies have focused on editing key genes across diverse crops to counteract the adverse effects of climate change (Kumar and Pandab, 2025). In line with these efforts, Kaur et al. examine the damaging effects of climate change on key cereal crops wheat, rice, and maize, and highlight how CRISPR/Cas9 genome editing can be strategically applied to safeguard global food security. The authors trace the evolution of crop improvement from conventional breeding and genetic modification (GM) technologies to the precision offered by genome editing tools. Drawing upon trait improvement examples from all three major cereals, they detail how CRISPR/Cas9 has been utilized to enhance agronomic traits such as yield, tolerance to abiotic stresses (including drought, salinity, and heat), resistance to biotic stresses like diseases, and improvements in nutritional content. By targeting specific genes associated with stress responses and productivity in wheat, rice, and maize, the review highlights the potential of genome editing in developing climate-resilient crop varieties essential for sustaining agricultural productivity in an increasingly uncertain climate.

While Kaur et al. highlighted the application of genome editing in cereals, Mestanza et al. further emphasize its role in fruit, vegetable, and tuber crops, with significant potential for improving agriculture in Peru. In their review, Mestanza et al. highlight the advances in CRISPR/Cas9 genome editing and its wide-ranging applications across diverse crop types in Peru, with a particular focus on transgene-free approaches. They discuss how CRISPR technologies, in addition to cereal crops, have been successfully applied to improve fruit crops by enhancing traits such as disease resistance and shelf life; vegetable crops by optimizing stress tolerance and nutrient content; and tuber crops, especially potatoes, by addressing climate-related challenges such as heat stress, drought, and pest infestations. These innovations are particularly relevant to Peru’s diverse agroecosystems, where regions like the Andean face chronic drought affecting staple crops such as potato, maize, quinoa, and beans. The authors also emphasize biofortification as a promising strategy to combat chronic malnutrition, which remains a pressing concern in vulnerable rural populations. Central to these efforts is the adoption of transgene-free genome editing strategies, such as the direct delivery of ribonucleoprotein (RNP) complexes, and incorporation of tRNA-like sequence (TLS) motifs into the single guide RNA (sgRNA) of the CRISPR/Cas system which enable precise genetic modifications without introducing foreign DNA into the plant genome. These approaches are especially significant in Peru, where regulatory frameworks are cautious regarding genetically modified organisms (GMOs), and a national moratorium imposes strict biosafety provisions. By leveraging transgene-free techniques, researchers can navigate these regulatory barriers, facilitating the development of crop varieties that exhibit improved resilience to biotic and abiotic stresses, enhanced nutritional profiles, and reduced reliance on agrochemicals like pesticides. Mestanza et al. advocate for a collaborative approach, emphasizing that cooperation among scientists, legislators, and farmers is essential to creating a robust regulatory framework that encourages technological innovation while safeguarding environmental sustainability and public health. Such a framework would enable the responsible adoption of genome editing technologies, contributing to food security and sustainable agriculture across Peru’s challenging and varied agricultural landscapes. Notably, some countries now exempt SDN-1 or SDN-2 edits from GMO oversight, accelerating their adoption. India has recently joined countries such as the United States, Japan, Canada, China, and the Philippines in approving genome-edited crops, with its first such rice varieties now approved.

The programmable RNA-guided endonuclease toolkit is rapidly expanding, enabling significant breakthroughs in genome editing for scientific research and biotechnological innovation. For example, a small, programmable RNA-guided gene-editing system called TIGR-Tas (Tandem Interspaced Guide RNA-Targeting Systems) was recently reported to overcome key limitations of CRISPR (Faure et al., 2025). Unlike CRISPR, which requires PAM sequences to target genes, TIGR-Tas is PAM-independent, potentially broadening the targetable genome. Its compact size and dual-guide mechanism, which interacts with both DNA strands, may improve precision and reduce off-target effects compared to single-guide RNA systems. These innovations illustrate how continuous technological advancement is expanding the precision, scope, and accessibility of genome editing. While such tools offer potential solutions to food security and sustainability challenges, concerns persist regarding intellectual property monopolies and equitable access for smallholder farmers. Furthermore, given the rapid pace of technological innovation, rigorous evaluation of biosafety and ecological impacts is critical to ensure that genome-editing technologies are deployed safely and sustainably (Kumar, 2025).

## References

[B1] Bezner Kerr R. Hasegawa T. Lasco R. Bhatt I. Deryng D. Farrell A. (2022). “Food, fibre, and other ecosystem products,” in Climate change 2022: impacts, adaptation and vulnerability. Contribution of working group II to the sixth assessment report of the intergovernmental panel on climate change. Editors Pörtner H.-O. Roberts D. C. Tignor M. Poloczanska E. S. Mintenbeck K. Alegría A. (Cambridge, UK, New York, NY, USA: Cambridge University Press), 713–906. 10.1017/9781009325844.007

[B2] Faure G. Saito M. Wilkinson M. E. Quinones-Olvera N. Xu P. Flam-Shepherd D. (2025). TIGR-Tas: a family of modular RNA-guided DNA-targeting systems in prokaryotes and their viruses. Science 388, eadv9789. 10.1126/science.adv9789 40014690 PMC12045711

[B3] IPCC (2023). “Climate change 2023: synthesis report,” in Contribution of working groups I, II and III to the sixth assessment report of the intergovernmental panel on climate change. Editors Lee H. Romero J. (Geneva, Switzerland: IPCC), 35–115. 10.59327/ipcc/ar6-9789291691647

[B4] Kumar K. R. R. (2025). Plant genome-editing goes viral: balancing innovation and biosafety. Trends in Biotechnology. 10.1016/j.tibtech.2025.09.006 40393857

[B5] Kumar K. R. R. Pandab P. (2025). “CRISPR-based genome editing for enhancing crop resilience to climate change-induced abiotic stresses,” in Handbook of nature-based solutions to mitigation and adaptation to climate change. Editors Leal Filho W. Nagy G. J. Ayal D. Y. (Cham: Springer). 10.1007/978-3-030-98067-2_174-1

[B6] Qiao J. Zang Y. Gao Q. Liu S. Zhang X. Hu W. (2025). Transgene- and tissue culture-free heritable genome editing using RNA virus-based delivery in wheat. Nat. Plants 11, 1252–1259. 10.1038/s41477-025-02023-8 40562814

